# Prevalence of chronic respiratory disease symptoms and associated occupational risk factors among certified silicosis patients of Rajasthan

**DOI:** 10.3389/fpubh.2026.1927403

**Published:** 2026-09-10

**Authors:** Suresh Yadav, Pranali Ahir, Dinesh Singh Bhati

**Affiliations:** ICMR-National Institute of Health Research (NIHR), Jodhpur, Rajasthan, India

**Keywords:** chronic respiratory disease, occupational lung disease, restrictive lung disorder, chronic respiratory symptoms, Rajasthan, sandstone quarrying, silicosis

## Abstract

**Background:**

Silicosis is an irreversible, fibrotic occupational lung disease caused by the chronic inhalation of respirable crystalline silica dust, and persists as a significant source of morbidity among sandstone quarry workers in Rajasthan, India. Despite the prevalence of silicosis, there has been no comprehensive assessment of the symptom load and occupational risk factors of Chronic Respiratory Symptoms among certified silicosis patients in the Balesar sandstone area.

**Methods:**

A community-based cross-sectional study was conducted in 355 certified silicosis patients (mean age 50.4 ± 10.6 years; all were exclusively male) who had resided for a minimum of 2 years in Balesar Block, Jodhpur, Rajasthan. Respiratory symptoms were evaluated using the ATS-DLD questionnaire. Predictors with a univariate chi-square *p* < 0.25 were included in a multivariable binary logistic regression model (IBM SPSS v25) to determine the independent predictors of composite Chronic Respiratory Symptoms status.

**Result:**

The findings showed that 286/355 patients, 80.6%, had composite Chronic Respiratory Symptoms. The most prevalent symptoms were morning cough (80.0%), morning phlegm production (79.7%), and dyspnoea attributable to cardiopulmonary impairment (78.0%); wheeze was noted by 51.3% and nocturnal breathlessness by 30.4%. The average age of symptom start was 40.4 ± 8.6 years, compared to 50.4 ± 10.6 years at certification, indicating a delay of about 10 years. In logistic regression, a conventional 8-h daily continuous work shift emerged as the most potent independent predictor of chronic respiratory disease symptoms (AOR 6.53, 95% CI 1.74–24.51, *p* = 0.005), while residing 6–10 km from the quarry also shown a significant correlation (AOR 2.74, 95% CI 1.07–6.99, *p* = 0.035). The associations between age at diagnosis (31–50 years) and illiteracy were statistically significant in the model (*p* = 0.021 and *p* = 0.035, respectively).

**Conclusion:**

This is the first study conducted on certified silicosis patients, illustrating that 80.6% of Balesar Block’s certified silicosis patients had active Chronic Respiratory Symptoms. The independent predictors were continuous standard-shift exposure and intermediate residential distance from quarries, strengthening the rationale for stricter daily exposure limits, mandatory dust suppression, and initial active case diagnosis in this occupational group.

## Highlights

- What is already known about this topic?: Silicosis is a significant, incurable occupational lung disease affecting sandstone workers in Rajasthan, with prevalence rates between (37–79%), strong association with pulmonary tuberculosis. Rajasthan’s 2019 Pneumoconiosis Policy offers ex-gratia compensation, but national reviews reveal low overall reporting and coverage (1, 2).
- What this study adds: Among certified silicosis patients specifically (not just exposed workers), Chronic Respiratory Symptoms burden is very high (80.6%), Standard shift (8 h/day) daily continues exposure and intermediate residence distance from the quarry are independent predictors after adjustment, while distance, employment-age, and diagnosis-age associations reported in earlier required correction against the underlying regression output.
- Recommendations for policy / what findings imply: The policy need to be more robust: limiting maximum daily working hours and mandatory wet-drilling/dust-suppression in all quarry zones (active and operational), widening active surveillance to all residents within 10 km of quarries (spirometry, chest radiography) regardless of specific employment, enhancing TB-silicosis dual-screening at primary health centres, and speeding up compensation allocation subsequent to the onset of symptoms rather than after an extended diagnostic time frame.

## 1 Introduction

Silicosis is an occupational pneumoconiosis arising from sustained inhalation of fine respirable crystalline silica particles, mainly in the form of quartz. Pathologically, it is characterised by the progressive development of fibrotic silicotic nodules within the lung parenchyma, which coalesce over time into progressive massive fibrosis, eventually impairing gas exchange and leading to severe restrictive or mixed ventilatory defects (3–7).

According to the Global Burden of Disease (GBD) 2023 estimates, silicosis caused 796,083 cases, 10,971 deaths, and 348,293 DALYs, age-standardised prevalence rate of 9.87 per 100,000 population. India alone had 1,824 cases and 577 deaths, while Rajasthan, one of the worst-affected states, had 112 cases and 47 deaths (8).

A nationwide comprehensive review and meta-analysis of Indian cohorts revealed 39.87 per 100 silicosis cases, 40.99 per 100 TB cases among silicosis, 8.74 per 100 silico-tuberculosis cases, highlighting the country’s significant burden (9).

Silicosis is an incurable condition causing irreversible lung scarring, progressing to fatal restrictive lung dysfunction. Pulmonary damage progresses even after silica exposure ceases leading to declining pulmonary function, respiratory failure, cor pulmonale, and mycobacterial infection (3, 10).

Chronic respiratory symptoms act as an early clinical marker of restrictive lung disease; patients with a restrictive spirometry pattern report significantly higher symptom burden, dyspnoea scores, and poorer quality-of-life scores, flagging functional impairment even before formal spirometric confirmation (11–13). This justifies their relevance in silicosis, an established restrictive occupational lung disease, where symptom surveillance can detect functional deterioration independent of, and often preceding, formal diagnostic confirmation.

Rajasthan’s sandstone belt, including Jodhpur, Barmer, and Jaisalmer, has consistently reported some of India’s highest silicosis prevalence rates, ranging from 8 to 52% (1, 14). These high-burden districts correspond in exposure patterns, workforce demographics, and mining practices with the Balesar Block of Jodhpur district, a centre of manual and mechanised sandstone extraction. Workers recruited from this region tend to be representative of the broader determinants of Chronic Respiratory Symptoms in silicosis patients (2, 9, 15–24).

Accordingly, this study aimed to (i) describe the sociodemographic and occupational profile of certified silicosis patients in The Balesar Block; (ii) estimate the prevalence of Chronic respiratory *symptoms* using the ATS-DLD questionnaire; and (iii) identify the occupational factors independently associated with Chronic respiratory *symptoms* presence through binary logistic regression, following systematic univariate screening as recommended for multivariable model building.

## 2 Methods

### 2.1 Study design and setting

**Figure 1:**
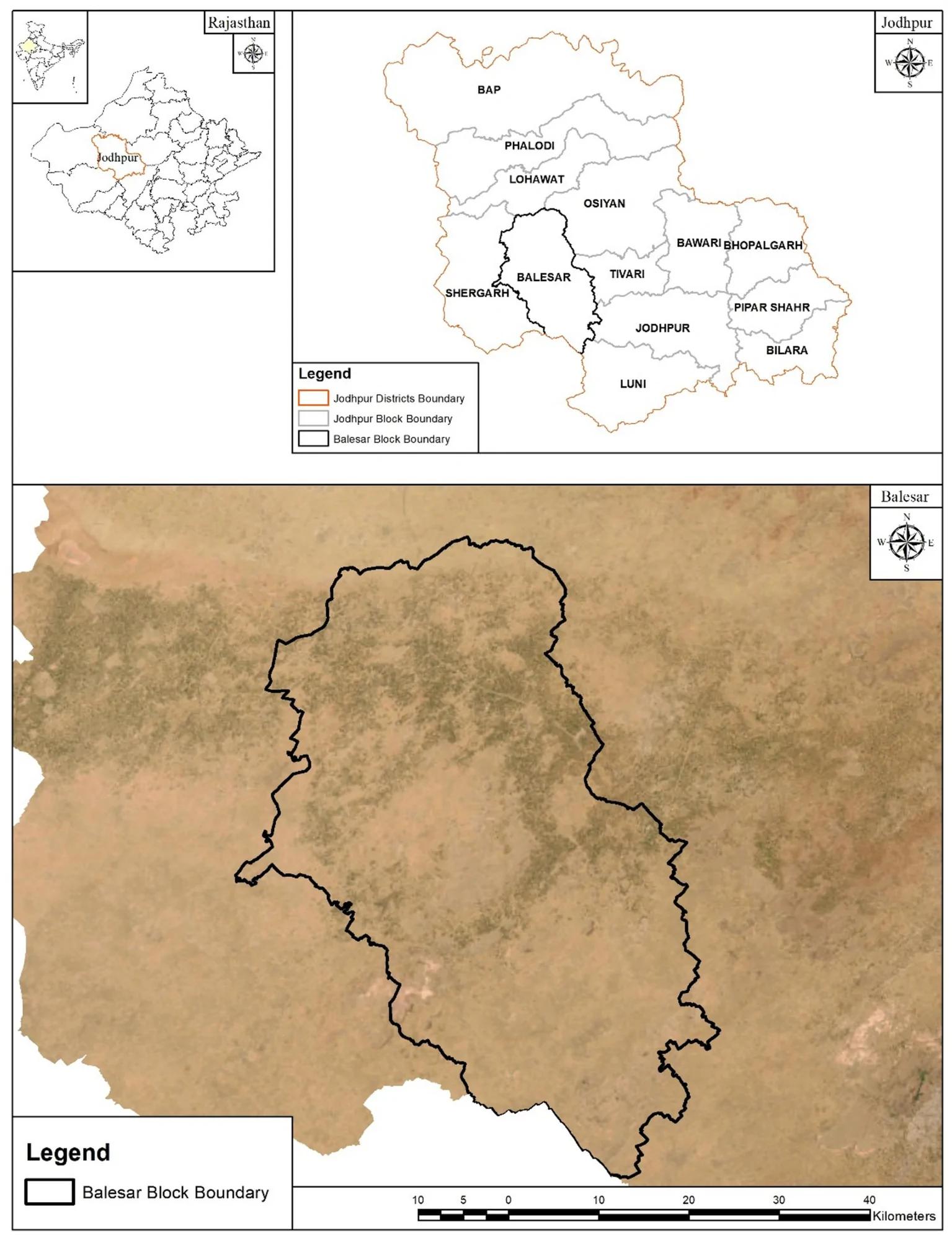
Map showing the study area of Balesar block.

This Community-based cross-sectional analytical research was conducted in Balesar Block, Jodhpur, Rajasthan, India. According to the 2011 Census of India, Balesar has 271 villages and an estimated population of 234,888. The study was conducted in Balesar Block, an administrative block of the Jodhpur district, Rajasthan, India (Figure 1).

Jodhpur, situated in the western part of the state of the Thar Desert, is characterised by a hot arid climate, low and erratic rainfall and the physiography of alluvial plains, sandstone formations and escarpments. The district is known for its large sandstone reserves and has one of the highest concentrations of sand stone quarries in the desert belt of Rajasthan. Sandstone mining and processing are major economic activities that employ a large segment of the local population. The Balesar Block is located at approximately 29°39′31″N latitude and 72°79′44″E longitude at an elevation of 250–300 m above the mean sea level (MSL). The block consists mainly of rural villages situated near sandstone quarries and stone processing units. Thus, a significant segment of the population is engaged in quarrying and associated activities resulting in long term occupational exposure to respirable crystalline silica dust and an increased risk of silicosis and other chronic respiratory diseases.

### 2.2 Study population and sampling size and approach

**Figure 2:**
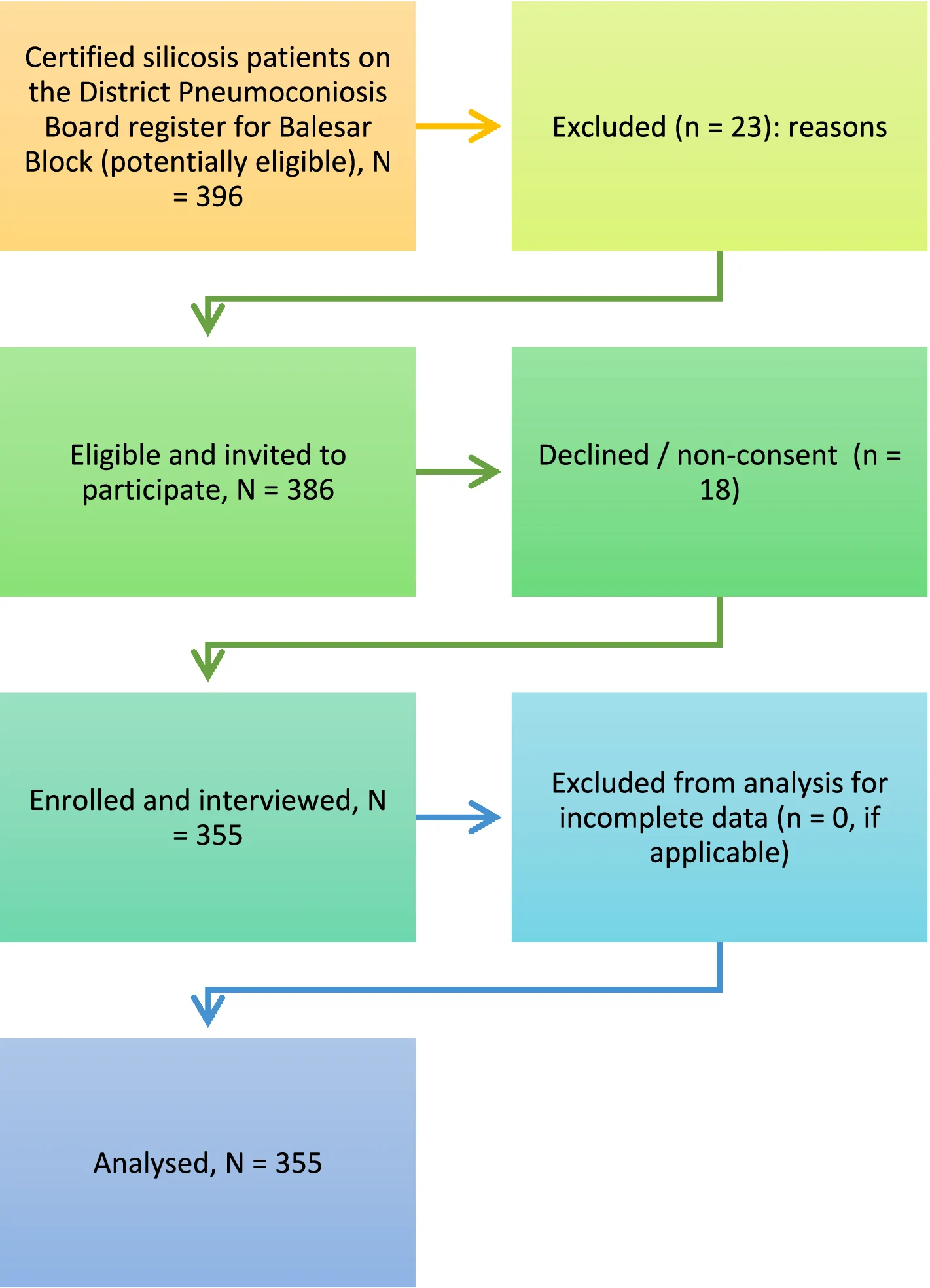
Flow diagram of participant enrolment.

The District Pneumoconiosis Board’s certified-case registry and village-level house-to-house enumeration by trained field investigators identified unlisted patients, ensuring near-complete coverage of the certified patients in the block. A participant flow chart shown in Figure 2.

### 2.3 Sample size

The sample size was established using Cochran’s method for prevalence estimates in cross-sectional studies: Where: n = minimum sample size; Z = 95% confidence level standard normal deviation (1.96); P = predicted prevalence of the outcome of interest; d = 5% absolute precision (margin of error).

The expected prevalence (P) used for the *a priori* calculation was 37.3% from a community-based cross-sectional survey of silicosis and respiratory morbidity among sandstone mine workers in Jodhpur district, Rajasthan, the closest setting-specific estimate to the Balesar (Jodhpur) study population since it is district-specific (Jodhpur) and occupationally matched (sandstone mining workers), this source provides the most defensible *a priori* P for this research group (1).

$$n=Z^{2}\times P\;(1-P)/d^{2}$$

$$\begin{array}{l} n={\left(1.96\right)}^{2}\times 0.373\times 0.627/{\left(0.05\right)}^{2}=\\ 3.8416\times 0.2339/0.0025\approx 359–360. \end{array}$$

10% added for anticipated non-response, giving n ≈ 396.

The current study enrolled all traceable certified silicosis patients who met the eligibility criteria from 271 villages in the Balesar Block. Since every participant in this study has a District Pneumoconiosis Board certified diagnosis of silicosis, symptom ascertainment entails identifying respiratory morbidity in diagnosed population. To avoid randomly selecting a sub-sample, the total-population (census) was approached. A sample size of 355 was determined to be optimal because it closely approximated the calculated limit.

### 2.4 Sampling technique

We enrolled all eligible Balesar Block silicosis patients using purposive (census) sampling. The records were collected from the District Pneumoconiosis Certified Silicosis Board at Jodhpur and the Balesar Samaj Kalyan Vibhag pension board register to ensure no cases were missing. These 2-board data were utilised to detect and compile the research area’s silicosis case list, facilitating case detection and recruitment. To locate certified patients in areas, field investigators used a systematic, block-wise house-to-house enumeration of Balesar tehsil’s 271 villages, hamlets (dhani, bas, bera, mohalla), and residential clusters, including Belwa, Durgawata, Devnagar, Jiya Bera, Rajgarh, Khari Beri, Chauthpura, Judiya, Junawas, Tolasar Charnan, Bhatelai Purohitan, Duggar, and Devatu This approach ensured near-complete geographic and community-level coverage, reduced selection bias from under-enumeration of remote or underprivileged hamlets, and enabled consent and interviewing all of the eligible sample to fulfil inclusion criteria.

Inclusion criteria

- Officially certified silicosis case by the District Pneumoconiosis Board.
- Minimum 2 years’ continuous residence in the Balesar Block at the time of the survey.
- History of sandstone-mining work or documented silica-dust exposure, irrespective of age at exposure.
- Age ≥18 years at the time of the interview.
- Willing and able to provide written informed consent.

Exclusion criteria

- Immunocompromised individuals.
- Patients unable/unwilling to provide informed consent.
- Residents who could not be traced or had migrated permanently out of the block.

### 2.5 Data collection

Our study enrolled patients from Jodhpur’s District Pneumoconiosis Board’s certified-case registry. The silicosis certification process has to be carried out in a series of steps: The District Pneumoconiosis Board evaluated and certified suspect silicosis patients after screening was done at the Community Health Centre (CHC). Three members of the Board, a radiologist, a chest physician, and a general practitioner, convene to evaluate the radiograph and clinical findings collaboratively before certification. The ILO International Classification of Radiographs of Pneumoconiosis mandates a small-opacity profusion category of 1/0 or greater (ILO standard films) for a positive silicosis case certification decision. The certificate lists a category and zone distribution. If the diagnostic criteria were fulfilled, silicosis was diagnosed and reported to the district authorities. The Centralised Pneumoconiosis Fund, supported by BOCW Welfare Board, DMFTs, and State Government, provided financial aid to eligible patients. In order to maximise efficiency and avoid human error, the entire process was conducted online (2). We further cross-verified the identified cases using the Balesar Samaj Kalyan Vibhag pension board register to ensure that no eligible silicosis cases were missed.

Since chest radiographs were classified against the ILO pneumoconiosis protocol rather than functional or symptom-based criteria, the records provide limited data on patients’ chronic respiratory symptoms burden after diagnosis Hence we Implemented a validated symptom instrument, the American Thoracic Society Division of Lung Diseases (ATS-DLD) questionnaire (25), on this radiologically confirmed population enable the study to assess the clinical progress of silicosis beyond certification, rather than re-estimating prevalence, which has already been extensively reported at state and national levels.

However, the Hindi version of the ATS -DLD was not available; hence, it was translated into Hindi and back-translated into English to ensure linguistic and conceptual equivalence (26). This translated questionnaire was tested in a separate Hindi-speaking community in Jodhpur with similar cultural characteristics, outside the main study population, to assess the clarity and comprehensibility of the questions. Participants from different age groups were included in the testing to ensure that the questions were understandable across the age spectrum. Data collectors were trained to administer the questionnaire consistently, particularly for potentially difficult respiratory symptoms, e.g., wheeze, etc. The same trained data collectors subsequently administered the questionnaire during the main study data collection.

Occupational history & sociodemographic (age, sex, caste, religion, education, marital status, and residence type), Clinical characteristics, healthcare utilisation, and compensation status were also collected through a structured questionnaire.

Occupational history was primarily self-reported, as no centralised quarry-level employment registry exists for informal sandstone-mining labour in the block. Mining is the second most common occupation in Rajasthan after agriculture, and in drought years, mining becomes primary. Regular sandstone quarry workers, small farmers, agricultural labourers, and others dependent on agriculture work in quarries after the kharif cropping season and during periods of drought. Agriculture in this region is predominantly rain-fed and follows a single-cropping pattern, with the kharif season generally extending from July to September (27).

Participants were asked their age at commencement of mine work, the specific mining/processing activity performed (drilling, cutting, loading, crushing), and any periods of discontinuation (e.g., due to illness, migration, or seasonal work). Cumulative occupational exposure duration was calculated as the sum of all discrete periods of active mining/processing work (current age minus age at start, minus any self-reported non-working intervals ≥6 months), rather than simple calendar-year span, to avoid overestimating true dust-exposure time. Data were collected between January 2021 and May 2021 across all 271 villages, which were visited sequentially in a block-wise at Balesar, Jodhpur district, Rajasthan.

All interviews were conducted face-to-face at the participant’s residence; no telephonic or proxy interviews were used. Each interview was conducted in Hindi by a trained field investigator after written informed consent, typically lasting 30–40 min, and supervised field visits were used for quality checks on a random subset of completed interviews.

### 2.6 Variables

The primary outcome was a composite of chronic respiratory symptoms, defined using standardised case-definitions from the ATS-DLD-78 questionnaire (25), not participants’ subjective interpretation. Chronic cough, was cough on most days for ≥5 consecutive months/year (Q1-E); chronic phlegm required the same pattern for ≥3 months/year (Q2-E), distinguishing pathological from transient, cold-related symptoms. Episodic cough/phlegm exacerbation required duration ≥3 weeks/year, recurring annually (Q3-A–B). The wheeze item (Q4-A) and nocturnal-symptom probe were administered with an added 12-month recall boundary “wheezing or whistling in your chest at any time in the last 12 months” consistent with the IUATLD Bronchial Symptoms Questionnaire and the European Community Respiratory Health Survey (ECRHS), which modified the ATS-DLD’s open-ended wheeze wording for improved recall accuracy and comparability (28, 29). This convention was later adopted by ISAAC, BOLD, and NHANES (30, 31). Chronic Respiratory symptoms were coded binary: symptoms present if ≥1 of 12 items was positive (32), else absent (Supplementary Table 1).

The predictor factors were current age group, mining job experience (years), daily working hours, type of mining process, distance of residence from the quarry, age at start of mine work, caste, religion, education level, and TB treatment history. Quantitative variables were categorised into clinically significant groups based on previous research and validated by analysing frequency distributions. Definitions added to Supplementary Table 2.

### 2.7 Operational definition

Chronic respiratory symptoms encompass persistent or recurrent manifestations of the respiratory tract chronic cough, phlegm production, wheezing, shortness of breath, and chest tightness that commonly arise from prolonged exposure to occupational or environmental respiratory hazards and often signal underlying chronic respiratory disease rather than transient illness (33).

In large population-based pulmonary surveys, chronic respiratory symptoms are operationally defined and measured as a composite of chronic cough, chronic phlegm, wheezing, and dyspnoea, assessed via standardised questionnaires and linked independently to reduced pulmonary function and poorer health-related quality of life, regardless of formal disease diagnosis (34).

### 2.8 Statistical analysis

Data were analysed using IBM SPSS Statistics version 25. The descriptive statistics included frequencies, percentages, means, and standard deviations. The normality of continuous variables was determined using the Kolmogorov–Smirnov and Shapiro–Wilk tests. Bivariate Pearson chi-square tests were used to assess each potential predictor and the composite Chronic Respiratory symptoms results. To avoid missing crucial predictors, variables with a liberal univariate screening threshold of *p* < 0.25 were maintained for multivariable modelling (35). Statistically relevant factors identified in prior studies were retained irrespective of their bivariate *p*-value. Multivariable binary logistic regression analysis was performed to identify factors associated with Chronic Respiratory symptoms. Adjusted odds ratios (AORs) with 95% confidence intervals were calculated. Categorical predictors were coded using the indicator with the greatest exposure or clinically significant reference category. Adjusted odds ratios (OR) and 95% confidence intervals (CI) are shown. A two-tailed *p*-value of less than 0.05 was used to determine statistical significance for the final model (35).

### 2.9 Bias reduction

Several measures were incorporated to limit information bias: (i) use of the standardised, pre-tested ATS-DLD-78 instrument rather than self-reported symptom; (ii) structured training and inter-rater calibration of all field investigators before data collection (iii) forward translation of items into Hindi followed by back-translation and pretesting in a non-study village to confirm conceptual equivalence; (iv) cross-checking of reported symptom-onset timing against available Pneumoconiosis Board records and prior treatment history where such records existed, to reduce recall drift; and (v) interviewers were not informed of the study’s specific hypotheses regarding occupational predictors, to reduce observer expectancy effects. To ensure total coverage throughout all 271 villages, listing all certified silicosis patients in the block rather than a select group reduced the selection bias. To reduce recall bias, we checked participant-reported age at symptom onset and job history against certification and treatment data. Using the standardised, validated ATS-DLD questionnaire delivered by qualified field investigators in Hindi and training all interviewers before data collection minimised information bias.

### 2.10 Missing data

Missing data were minimal across sociodemographic and occupational, chi-square, and logistic regression variables; the specific counts are footnoted in tables where applicable.

### 2.11 Ethical considerations

Institutional ethical clearance was obtained from the Institutional Ethics Committee (IEC) of ICMR–NIHR (formerly ICMR–NIIRNCD), Jodhpur, during its 16th IEC meeting held on January 11, 2020. The approval certificate was issued on January 20, 2020 in accordance with ICMR guidelines. Written informed consent was obtained from all participants before enrolment. Participant confidentiality was maintained, and the data were stored in password-protected files accessible only to the research team.

## 3 Results

### 3.1 Sociodemographic profile

Data from 355 patients with certified silicosis were analysed. The research population surveyed was exclusively male (*n* = 355), illustrating the gender composition of the sandstone quarrying in this area. The predominant age group of participants was 40–64 years (*n* = 238, 67.0%), whereas 22.5% were aged above 65 years. Scheduled Castes were the predominant caste group (*n* = 169, 47.6%), followed by Other Backwards Classes (*n* = 155, 43.7%). The majority of participants (95.5%) identified as Hindu. The marriage rate was 86.2%. Illiteracy was prevalent, with 61.4% of the sample lacking formal schooling, and 18.0% were literate just at the basic read-and-write level. Residential occupancy was almost evenly split between rental (50.7%) and owner-occupied (48.7%) housing, mostly located near mining areas. The detailed sociodemographic data are presented in Table 1.

**Table 1:** Sociodemographic characteristics of certified silicosis patients, Balesar block, Jodhpur, Rajasthan (*n* = 355).

Variable	Frequency (*n* = 355)	Percent (%)
Age group (Current)		
18–39 years	37	10.4
40–64 years	238	67.0
>65 years	80	22.5
Caste		
General	24	6.8
Other backward classes	155	43.7
Scheduled castes	169	47.6
Scheduled tribes	6	1.7
Others	1	0.3
Religion		
Hindu	339	95.5
Muslim	14	3.9
Christian	1	0.3
Sikh	1	0.3
Marital status		
Married	306	86.2
Unmarried	11	3.1
Divorced	9	2.5
Separated	14	3.9
Widow	15	4.2
Education level		
Illiterate	218	61.4
Read and write only	64	18.0
Primary school	41	11.5
Middle school	28	7.9
High school	4	1.1
Residence type		
Rented	180	50.7
Self-owned	173	48.7
Homeless	2	0.6

No missing value.

### 3.2 Occupational characteristics

The majority of the mining lease sample consisted of participants with over 21 years of mining experience (*n* = 234, 65.9%), illustrating the persistent exposure conditions characterising this workforce. Although most miners started working between the ages of 16 and 25 (*n* = 251, or 70.4%), more than a quarter started at or below the age of 15, suggesting that occupational silica exposure began at a young age (27.6%). On a daily basis, standard shift (8 h/day) daily continued exposure 167(47%). The most prevalent mining process consisted of material handling and integrated extraction activities (57.2%). Significantly, 76.6% of mine workers did not employ dust-inhalation protection. The distance from the quarry was unequally distributed among the three groups. Table 2 summarises the occupational characteristics.

**Table 2:** Occupational characteristics of certified silicosis patients, Balesar block, Jodhpur, Rajasthan (*n* = 355).

Variable	Frequency (*n* = 355)	Percent (%)
Age at which mine work commenced		
≤15 years	98	27.6
16–25 years	251	70.4
≥26 years	7	2.0
Total mining work experience		
≤10 years	12	3.4
11–20 years	109	30.7
>21 years	234	65.9
Working hours per day		
Less than standard shift (<8 h/day)	74	20.8
Standard shift (8 h/day)	167	47.0
Irregular shift/on-call/alternate day >48 h/week	114	32.1
Type of mining process involvement		
Manual Extraction and Primary Breaking	41	11.5
Drilling and Blasting (Mechanised)	70	19.7
Material Handling and Combined Extraction	203	57.2
Stone Processing, Dressing and Finishing	41	11.5
Use of dust inhalation preventive measures (PPE)		
No	272	76.6
Yes	83	23.4
Residence distance from quarry/mine		
1–5 km	120	33.8
6–10 km	119	33.5
>11 km	116	32.7

PPE, Personal Protective Equipment, No missing value.

### 3.3 Clinical and disease characteristics

The average age at official silicosis diagnosis was 50.37 years (SD = 10.59; range: 26–84 years), with almost equal distributions in the 31–50 years (49.9%) and above 51 years (49.0%) age-at-diagnosis categories. A clinical latency estimated to be more than 10 years was observed between the onset of symptoms and formal certification, as the mean age at first symptom onset was 40.39 years (SD = 8.65; range: 20–60 years). The majority of symptom onsets (74.1%) occurred in the 31–50-year age demographic. In terms of healthcare utilisation, 21.4% of patients completed four or more courses of anti-tuberculosis therapy, and 48.7% of participants had undergone 1 to 3 chest X-ray assessments overall, demonstrating significant co-morbidity between silico-tuberculosis in this population. The majority of participants (89.3%) have received government silicosis compensation at least once throughout their lives, with 37.7% receiving ₹1,00,000 and 58.6% receiving ₹2,00,000. The clinical and compensation data are presented in Table 3.

**Table 3:** Clinical characteristics, healthcare utilisation, and compensation status of certified silicosis patients (*n* = 355).

Variable	Frequency (*n* = 355)	Percent (%)
Age at silicosis diagnosis (years)—Mean: 50.37 ± 10.59; Range: 26–84		
≤30 years	4	1.1
31–50 years	177	49.9
≥51 years	174	49.0
Age at first silicosis symptom onset (years)—Mean: 40.39 ± 8.65; Range: 20–60		
≤30 years	61	17.2
31–50 years	263	74.1
≥51 years	31	8.7
Chest X-ray examinations		
Low frequency: 1–3 times	173	48.7
Moderate frequency: 4–6 times	121	34.1
High frequency: ≥7 times	61	17.2
Anti-tuberculosis treatment courses taken		
Low/Minimal: 0–1 course	51	14.4
Moderate: 2–3 courses	228	64.2
Multiple/Recurrent: ≥4 courses	76	21.4
Sputum examinations performed		
Low: 1–3 times	161	45.4
Moderate: 4–6 times	141	39.7
Frequent: ≥7 times	53	14.9
Silicosis compensation timing		
Received during lifetime	317	89.3
Received after death (to family)	38	10.7
Compensation amount received (INR)		
₹1,00,000	134	37.7
₹2,00,000	208	58.6
₹5,00,000	10	2.8
Other amounts	3	0.8

INR, Indian Rupee. TB, Tuberculosis. SD, Standard Deviation, No missing value.

### 3.4 Prevalence and pattern of chronic respiratory symptoms

Among the 355 participants, 286 (80.6%) had at least one chronic respiratory symptom, while 69 (19.4%) reported no symptoms. Morning cough in winter was the most frequently reported symptom (80.0%), followed by morning phlegm production in winter (79.7%) and breathlessness due to heart or lung disability (78.0%). Cough for 5 consecutive months or more was reported by 74.1%, while 67.3% reported phlegm production for ≥3 months per year. Increasing cough and phlegm lasting ≥3 weeks annually was reported by 68.7%. Wheezing in the past 12 months was reported by 51.3%, while 31.3% experienced attacks of shortness of breath with wheezing and 30.4% reported nocturnal shortness of breath. Overall, the findings indicate a high burden of respiratory symptoms in the study population (Table 4).

**Table 4:** Prevalence of individual chronic respiratory by ATS-DLD questionnaire items (*n* = 355).

Chronic respiratory symptoms (ATS-DLD questionnaire)	Frequency (n)	Percent (%)
Cough symptoms		
Morning cough in winter	284	80.0
Cough for 5 consecutive months or more	263	74.1
Increasing cough and phlegm lasting for 3 weeks or more each year	244	68.7
Cough occurring in multiple episodes per year	215	60.6
Cough overproduction (winter)		
Day	237	66.8
Night	42	11.8
Day & Night	5	1.4
No cough	71	20.0
Phlegm production		
Morning phlegm production in winter	283	79.7
Phlegm production ≥3 months per year	239	67.3
Phlegm overproduction (winter)		
Day	235	66.2
Night	44	12.4
Day & Night	6	1.7
No phlegm	70	19.7
Breathlessness		
Breathlessness (due to heart or lung disability)	277	78.0
Wheezing		
Wheezing in the past 12 months	182	51.3
Attacks of shortness of breath with wheezing	111	31.3
Normal breathing between wheezing attacks 2 or more such episodes	110	31.0
Nocturnal shortness of breath in past 12 months	108	30.4
Composite chronic respiratory symptoms outcome (≥1 yrs symptom present)		
Chronic Respiratory Symptoms Present	286	80.6
Chronic Respiratory Symptoms Absent	69	19.4

ATS-DLD, American Thoracic Society Division of Lung Disease, No missing value.

### 3.5 Bivariate chi-square analyses

Bivariate chi-square analyses were conducted between the composite chronic respiratory symptom’s outcome and all predictor variables. Associations between each predictor and composite chronic respiratory symptoms were evaluated using Pearson chi-square cross-tabulations. Although the correlation was not significant (*p* = 0.111), Chronic Respiratory Symptoms was more common among miners who lived 6–10 km from quarries (102; 33.5%) than non-Chronic Respiratory Symptoms miners (17; 24.6%; *p* = 0.111) Age at diagnosis was marginally significant (31–50 years: 145; 50.7% Chronic Respiratory Symptoms; *p* = 0.094). Standard-shift workers comprised 163 (57.0%) Chronic Respiratory Symptoms patients compared to 4 (5.8%) non-cases (*p* < 0.001), indicating a high correlation between daily working hours and Chronic Respiratory Symptoms. Despite limited data, education was maintained (illiterate: 173; 60.5%; *p* = 0.232). Age at beginning of mining (*p* = 0.233). Work done by Miners (*p* = 0.914) was eliminated, whereas variables with *p* < 0.25 were included in the logistic bivariate regression. Duration of mining experience was significantly associated with Chronic Respiratory Symptoms (*p* = 0.001), with >21 years of work experience accounting for 196 (68.6%) CRD cases compared with 38 (55.1%) non-Chronic Respiratory Symptoms participants. The results of the bivariate analysis are presented in Table 5.

**Table 5:** Cross-tabulation chi-square analysis: association of predictor variables with composite chronic respiratory symptoms status (*n* = 355).

Variable	Chronic respiratory symptoms		*p*-value
	Yes	No	
Residence distance from the quarry			
>11 Kms	94 (32.9%)	22 (31.9%)	0.111
6–10 Kms	102 (33.5%)	17 (24.6%)	
1–5 Kms	90 (31.5%)	30 (43.5%)	
Age at which the miner’s started mine work			
= < 15 Years	77 (30.4%)	21 (26.9%)	0.233
16–25 Years	205 (66.7%)	46 (71.3%)	
> = 26 Years	5 (2.9%)	2 (1.7%)	
Age at which silicosis was diagnosed			0.094
<=30 Year	3 (1.0%)	1 (1.4%)	
31–50 Years	145 (50.7%)	32 (46.4%)	
= > 51 Years	138 (48.3%)	36 (52.2%)	
Daily working hours in mine-related work			
Less than standard shift (<8 h/day)	21 (7.3%)	53 (76.8%)	<0.001
Standard shift (8 h/day)	163 (57.0%)	4 (5.8%)	
Irregular shift/on-call/alternate day >48 h/week	102 (35.7%)	12 (17.4%)	
Education of miners			
Illiterate	173 (60.5%)	45 (65.2%)	0.232
Read and write	53 (18.5%)	11 (15.9%)	
Primary school	32 (11.2%)	9 (13.0%)	
Middle school	25 (8.7%)	3 (4.3%)	
High school	3 (1.0%)	1 (1.4%)	
Work done by miners			
Manual Extraction and Primary Breaking Operations	36 (12.6%)	5 (7.2%)	0.914
Drilling and Blasting Operations (Mechanised Extraction)	58 (20.3%)	12 (17.4%)	
Material Handling and Combined Extraction Operations	153 (53.5%)	50 (72.5%)	
Stone Processing, Dressing and Finishing Operations	39 (13.6%)	2 (2.9%)	
Work experience by miners			
<=10 Years	5 (1.7%)	7 (10.1%)	0.001
11–20 Years	85 (29.7%)	24 (34.8%)	
>21 Years	196 (68.6%)	38 (55.1%)	

Values are n (row %). Chi-square *p*-values refer to the overall test for the variable. * *p* < 0.05, No missing value.

### 3.6 Multivariable binary logistic regression analysis showing adjusted odds ratios for factors associated with chronic respiratory symptoms

Multivariable binary logistic regression analysis was performed with the presence of composite Chronic Respiratory Symptoms as the dependent variable. Standard shift (8 h/day) daily continued exposure showed the strongest and most consistent independent association with Chronic Respiratory Symptoms (AOR = 6.53, *p* = 0.005), Patients residing at 6–10 km from the quarry had higher odds (AOR 2.74, 95% CI 1.07–6.99, *p* = 0.035). Risks of silicosis diagnosis at 31–50 years were elevated Chronic Respiratory Symptoms (AOR 1.38, 95% CI 0.63–3.04, *p* = 0.42). Education level below secondary school was associated with higher odds (AOR 1.32, 95% CI 0.02–5.63, *p* = 0.35). Residences >11 km, younger age during mine labour, diagnosis ≤30 years, and read-and-write education were not significant. Multiple estimates with wide confidence ranges indicate imprecision from narrow reference groups. The detailed logistic regression results are presented in Table 6.

**Table 6:** Multivariable binary logistic regression analysis showing adjusted odds ratios (AORs) for factors associated with chronic respiratory symptoms.

Variable / Category	Frequency	Percentage (%)	AOR	95% CI	*p*
Residence distance from quarry (ref: 1–5 km)					
>11 km	116	32.7	2.06	0.82–5.14	0.123
6–10 km	119	33.5	2.74	1.07–6.99	0.035*
Age at which the miner’s started mine work (ref: ≥26 yrs)					
≤15 years	98	27.6	8.15	0.65–101.72	0.103
16–25 years	251	70.4	9.95	0.86–115.01	0.066
Age at which silicosis was diagnosed (ref: ≥51 yrs)					
≤30 years	4	1.1	0.54	0.01–46.69	0.785
31–50 years	177	49.9	1.38	0.63–3.04	0.421
Daily working hours in mine related work (ref: irregular shift/on-call/alternate day >48 h/week)					
Less than standard shift (<8 h/day)	74	20.8	0.04	0.02–0.10	<0.001*
Standard shift (8 h/day)	167	47.0	6.53	1.74–24.51	0.005*
Education of Miners (ref: High School)					
Illiterate	218	61.4	1.32	0.02–5.63	0.355
Read & Write only	64	18.0	1.49	0.03–9.46	0.639
Primary School	41	11.5	0.21	0.01–4.25	0.309
Middle School	28	7.9	0.34	0.01–9.41	0.527
Constant	—	—	1.43	—	0.853

*p < 0.05. AOR, Adjusted Odds Ratio; CI, Confidence Interval. *n* = 355. Model variables entered: residence distance, age at starting mine work, age at silicosis diagnosis, daily working hours, education level, No missing values.

**Figure 3:**
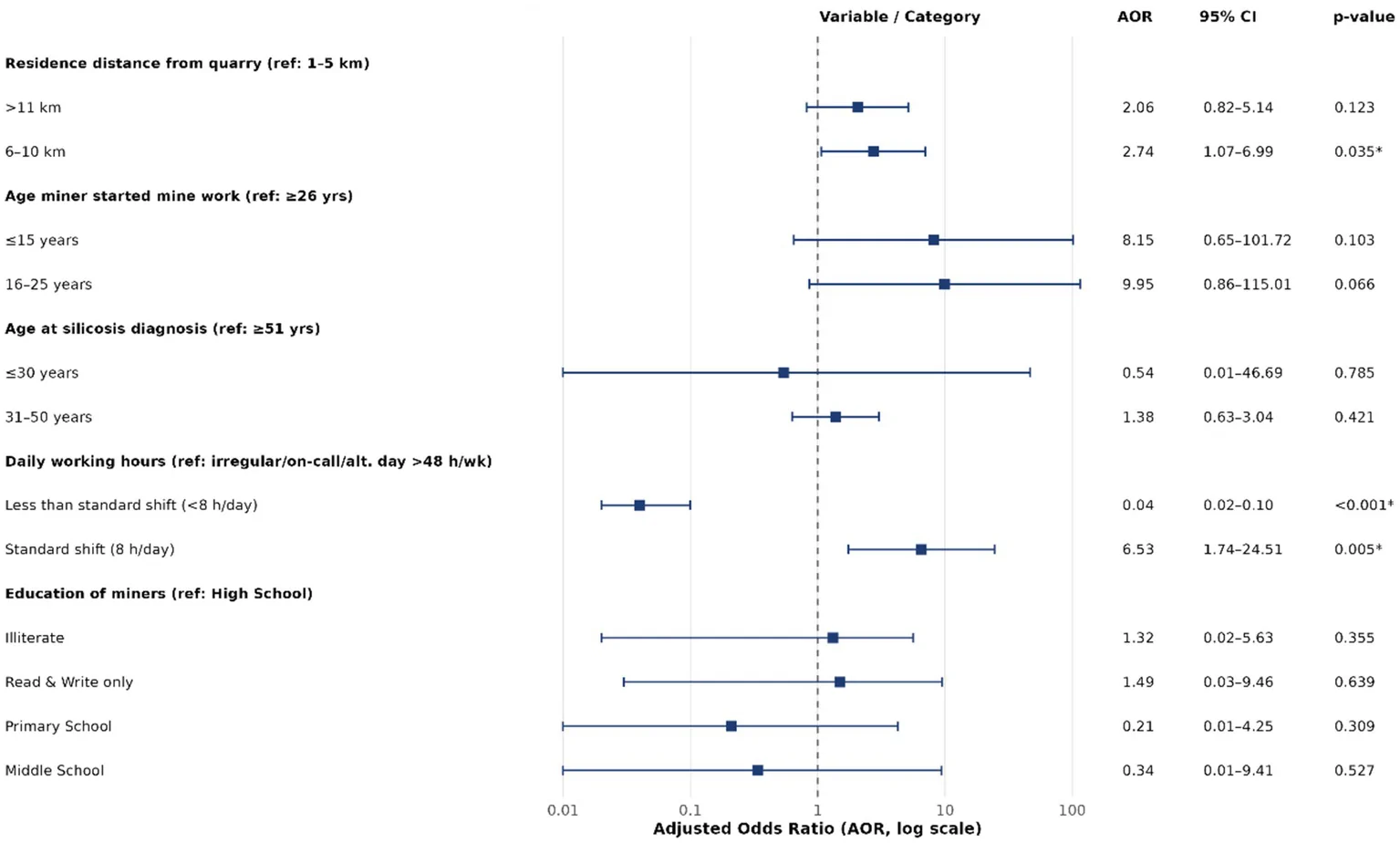
Forest plot of adjusted odds ratios for factors associated with chronic respiratory symptoms among stone quarry miners.

Figure 3 Adjusted odds ratios (AORs) of factors associated with chronic respiratory symptoms among certified silicosis patients. The likelihood of chronic respiratory symptoms among miners living 6–10 km from quarries was significantly higher than those living 1–5 km away (*AOR* = 2.74, 95% *CI*: 1.07–6.99; *p* = 0.035) after adjustment. Having an 8-h shift was also associated with increased odds of chronic respiratory symptoms (*AOR* = 6.53, 95% *CI*: 1.74–24.51; *p* = 0.005).

## 4 Discussion

The prevalence of silicosis across recent global and Indian literature remains substantial despite an overall decline in age-standardised burden (1, 2, 9, 14, 18, 19, 21, 23, 24, 36–42). A 2026 systematic review and meta-analysis of 162,449 mineral miners across 26 countries estimated a pooled global silicosis prevalence of 17%, rising to 20% among stone-sector workers specifically (37). Screening data from Australia’s stone industry found silicosis in 28.2% of workers undergoing secondary screening, at a median tenure of only 12 years (43). Within Rajasthan itself, district-level prevalence ranges from 8 to 52% depending on quarry intensity and screening technique, with the upper end concentrated in the most heavily mined sandstone belts (14).

This study’s findings extend the prevalence literature by showing 80.6% (286/355) of certified silicosis patients in Balesar Block had at least one chronic respiratory symptoms on the ATS-DLD instrument. Winter morning cough was the most prevalent symptom (80.0%, 284/355), followed by morning phlegm (79.7%, 283/355) and dyspnoea (78.0%, 277).

Silica inhalation is well established to trigger mucus hypersecretion and impaired mucociliary clearance, a mechanism driven by ciliary dysfunction and goblet-cell hyperplasia in chronically irritated airways (44), and consistent with morning cough being the most common individual symptom in this cohort (80.0%). Breathlessness attributable to cardiac or pulmonary dysfunction affected 78.0% of patients, suggesting substantial functional restriction and quality-of-life impairment. Wheezing (51.3%) was higher than typically reported in general Indian population samples and is compatible with silica-induced airway obstruction occurring independently of smoking status.

The Karauli district of Rajasthan revealed these findings: miners had lower rates of wheezing, dyspnoea, and haemoptysis, whereas 31% of ex-miners reported worsening respiratory problems as the reason they quit the mining sector (45). Breathlessness linked to cardiac or pulmonary dysfunction was (78%), suggesting profound functional restriction and quality of life impairment in this population (14). Wheezing (51.3%) was higher than in Indian general population samples and compatible with reports of silica-induced airways blockage irrespective of smoking status (36). In the US, 31.5% of retired coal miners had cough dyspnoea with radiographic disease progression (46). Former gold miners in South Africa experienced a higher prevalence of chronic respiratory symptoms and higher rates of tuberculosis (TB) screening years after leaving the mining industry (47).

Chronic Respiratory symptoms elevated moderately to severely in 37.2% of Chilean former miners (48). Similar post-exposure symptoms prevail in former miners in pooled artisanal-mining cohorts from 10 different countries (49). The pneumoconiosis estimations in South Africa’s cement manufacturing industry were far higher than those based on symptoms (50).

This study highlights Chronic Respiratory symptoms burdens as a clinically relevant marker rather than an inactive indicator of previous exposure. Since severity of symptoms tracks disease progression, it may guide the period of referrals for pulmonary rehabilitation, recommend spirometry or repeat imaging, and screen for tuberculosis, thus preventing acute exacerbations, hospitalisations, and premature death.

This reduces long-term treatment costs, disability compensation burden, and productivity loss, while improving quality of life and survival tasks that one-time radiographic certification cannot fulfil. The age at symptom onset (median 40 years) suggests that exposure levels in the Balesar sandstone sector are sufficient to produce symptomatic disease at an earlier age than in other silica-exposed occupations (40). Clinically significant is the 10-year interval between mean symptom start (40.4 years) and diagnosis (50.4 years). This indicates that workers may continue exposure without medical observation or reimbursement for a large part of the illness course before official certification.

The 27.6% of adults who started employment before 15 years old special concern because childhood-onset exposure significantly increases cumulative lifetime exposure and worsens silicosis pathologies (38).

The independent risk factor of Chronic Respiratory symptoms identified were, continuous 8-h daily exposure (AOR = 6.53, 95% CI 1.74–24.51, *p* = 0.005); associations with earlier age at mine-work commencement (AOR = 9.95, 95% CI 0.86–115.01, *p* = 0.066) and diagnosis silicosis at 31 years (AOR = 1.38, *p* = 0.421).

This significant exposure-duration finding is consistent with, evidence at multiple global studies like cumulative respirable silica dose is the most reproducible determinant of silicosis severity in pooled cohorts (18); in China multicentre occupational data 2,064 dust-exposed workers, exposure intensity independently predicted obstructive pulmonary dysfunction after adjustment for age and duration (51); and in a Rajasthan-specific occupational cohort, uninterrupted shift patterns were similarly linked to elevated respiratory symptom risk (36).

Other risk factor like Illiteracy (AOR = 1.32) and residence 6–10 km from the quarry (AOR = 2.74, 95% CI 1.07–6.99, *p* = 0.035) were also associated with Chronic Respiratory symptoms. The nearest residential band did not carry the highest odds; this is consistent with mining-belt studies of ambient dust drift, which document non-linear, topography- and wind-dependent dust settling rather than a simple decline with distance (21, 23).

Recurrent anti-TB treatment was significantly associated with Chronic Respiratory symptoms (*p* < 0.001), which is consistent with the widely recognised correlation between silicosis and tuberculosis, which has been reported in the Indian and global mining publications to enhance tuberculosis risk by 2.8–39 times (9, 18).

In the Rajasthan compensation scheme, only 58.6% of patients received the higher ₹2,00,000 tier, and 10.7% of families were compensated only after death. The finding that 97.5% of patients obtained certification through their self-initiative rather than employer or government-led screening raises concerns about the effectiveness of proactive surveillance in this area, highlighting a gap between policy intent and implementation despite centralised funding (2).

## 5 Conclusion

This research illustrates that 80.6% of Balesar Block certified silicosis patients had persistent respiratory symptoms. The data indicate substantial occupational susceptibility, with 65.9% having over 21 years of mining experience, 27.6% starting at ≤15 years, and 76.6% not using dust-protective measures. The risks of chronic respiratory disorder were greater for those who lived 6–10 km from the quarry and worked 8-h shifts (AOR 6.53). The symptom onset-to-certification timeframe suggests detection and treatment delays. In silica-exposed populations, comprehensive occupational assessment, early respiratory evaluation, exposure mitigation, and systematic TB screening are recommended. Cross-sectional design, purposeful census of certified cases, and lack of a comparator group limit temporal and causal findings. Future long-term research must involve quantified silica exposure, spirometry, radiological progression, and healthcare-access indicators. Strengthening workplace dust control and routine surveillance may enable earlier detection and reduce preventable respiratory disability in sandstone-mining communities.

### 5.1 Strengths and research gap

This study has several merits. There are limited studies with comprehensive data on all enrolled participants utilising a validated ATS-DLD symptom tool in a large cohort of certified silicosis patients in Rajasthan, an analysis performed using structured bivariate screening before logistic regression modelling.

The study’s main strength is that it covers a valid, underexplored evidence gap: state-run radiographic records certify the condition once and discontinue surveillance, but silicosis progresses due to ongoing inflammatory and fibrotic activity even after silica exposure ends, rather than solely via continued dust deposition (4).

In this study, persistent morning cough remains the most prevalent outcome even among workers who discontinued active quarry work. Repeatable symptom data therefore has direct clinical value beyond description: a rising or persistent symptom score in a certified, no-longer-exposed patient is an actionable trigger for referral, tuberculosis screening, or spirometry that can interrupt disease progression before severe irreversible lung damage.

### 5.2 Policy recommendations

Rajasthan’s 2019 Pneumoconiosis Policy already established certification boards, an online relief portal, and compensation financing (52), but coverage stops at the point of certification and excludes unregistered informal quarries where most exposure occurs.

Since silicosis remains incurable, policy must prioritise primary prevention through strict control of respirable crystalline silica exposure and mandatory workplace hygiene measures. Early diagnosis, patient counselling on exposure avoidance, and smoking cessation should be embedded into occupational health protocols to slow disease progression. Given the absence of validated anti-fibrotic or anti-cytokine therapies, research centre should prioritise funding for randomised controlled trials targeting silicosis pathophysiology (3). Mandatory reporting of new silicosis diagnoses to health departments and integration into national silica surveillance registries would strengthen outreach and targeted prevention strategies. Every diagnosis should trigger workplace hygiene audits, including wet-cutting technology and respiratory protective equipment.

Since in this study Chronic Respiratory symptoms data show deterioration continuing after workers leave mining, policy should mandate periodic, validated symptom questionnaires at primary health centres, bi-annual Chronic Respiratory symptoms check-ups, with worsening scores triggering automatic referral for spirometry, chest imaging, or tuberculosis screening. Integrating this into India’s existing TB-elimination infrastructure would transform silicosis management from a passive, one-time certification event to an active surveillance regular process, detecting silico-tuberculosis and functional decline before progression of severe irreversible damage. This would be a direct, low-cost extension of current policy rather than an alternative approach.

### 5.3 Limitations

This study has a few limitations; the cross-sectional design limits inferences regarding the trend of the observed associations. Several logistic regression estimates had large confidence intervals due to low reference-category numbers, which limited accuracy, especially for age at mine-work begin and education level. All subjects were certified silicosis patients; thus, the findings cannot be applied to non-certified sandstone workers in the community. Since this exposure-response analysis lacked spirometry data and objective ambient dust measurements, symptom findings could not be validated against lung function or exposure concentration. The study population was all male, which limited the scope of findings for female and paediatric groups exposed to mine dust. Further longitudinal research integrating spirometry and repeated post-exposure symptom evaluation were crucial to confirm the findings reported in this study.

## Data Availability

The original contributions presented in the study are included in the article/Supplementary material, further inquiries can be directed to the corresponding author.

## Author’s note

The research involved a community survey in a resource-limited area; therefore, patients or the public had no role in its design, conduct, or disclosure. The study findings were communicated to relevant government bodies through technical reports and policy briefs, and disseminated to the general population via digital media platforms, our institutional website, and mass media.
