# Supplementary material for: Prevalence of chronic respiratory disease symptoms and associated occupational risk factors among certified silicosis patients of Rajasthan
Source: Front Public Health. 2026 Sep 10;14:1927403. doi: 10.3389/fpubh.2026.1927403 (PMC13601359; doi:10.3389/fpubh.2026.1927403)
Supplement: Supplementary file 1 [file Table_1.docx]

Table 1. Detail of chronic respiratory disease (CRD) symptoms by ATS-DLD questionnaire

| **S. No.** | **Symptom Construct** | **ATS-DLD-78-A Question No.** | **Letter/No.** |
| --- | --- | --- | --- |
| 1 | Morning cough in winter | Question 1 – Cough | C |
| 2 | Cough for 5 consecutive months or more | Question 1 – Cough | E |
| 3 | Increasing cough and phlegm lasting ≥3 year | Question 3 – Cough | A |
| 4 | Cough occurring in multiple episodes per year | Question 3 – Cough | B |
| 5 | Cough overproduction – Day | Question 1 – Cough | D |
| 6 | Cough overproduction – Night | Question 1 – Cough | D |
| 7 | Cough overproduction – Day & Night | Question 1 – Cough | C + D |
| 8 | No cough | Question 1 – Cough | A (= No) |
| 9 | Morning phlegm production in winter | Question 2 – Phlegm | C |
| 10 | Phlegm production ≥3 months per year | Question 2 – Phlegm | E |
| 11 | Phlegm overproduction – Day | Question 2 – Phlegm | D |
| 12 | Phlegm overproduction – Night | Question 2 – Phlegm | D |
| 13 | Phlegm overproduction – Day & Night | Question 2 – Phlegm | C + D |
| 14 | No phlegm | Question 2 – Phlegm | A (= No) |
| 15 | Breathlessness (due to heart or lung disability) | Question 5 – Breathlessness | A |
| 16 | Wheezing in the past 12 months | Question 4 – Wheezing | A |
| 17 | Attacks of shortness of breath with wheezing | Question 4 – Wheezing | C |
| 18 | Normal breathing between wheezing attacks (≥2 episodes) | Question 4 – Wheezing | E |
| 19 | Nocturnal shortness of breath in past 12 months | Question 4 – Wheezing | A(3) |

**Operational Definitions**

Supplementary S1: Operational Definitions of Chronic Respiratory Symptoms (ATS‑DLD‑78‑A)

All respiratory symptom constructs used in this study were operationalized directly from the ATS‑DLD‑78‑A (Ferris, 1978) case‑definition logic embedded in the questionnaire's skip‑pattern structure, rather than from unqualified self‑report of a single "yes/no" item. Definitions were as follows:

Chronic cough: A positive response to "usually have a cough" (Q1‑A) and a positive response to "cough...on most days for 5 consecutive months or more during the year" (Q1‑E). Cough reported only in the context of an acute illness (e.g., a common cold), without meeting the ≥5‑month chronicity criterion, was coded symptom‑negative for this construct.

Chronic phlegm: A positive response to "usually bring up phlegm" (Q2‑A) and a positive response to "phlegm...on most days for 3 consecutive months or more during the year" (Q2‑E).

Recurrent cough‑and‑phlegm exacerbation: A positive response to episodes of increased cough and phlegm lasting ≥3 weeks, recurring at least once per year (Q3‑A–B).

Wheeze (past 12 months): A positive response to any of the three-wheeze sub‑items with colds, apart from colds, or most days/nights (Q4‑A1–3).

Recurrent wheeze attack with breathlessness: A positive response to "ATTACK of wheezing that has made you feel short of breath" (Q4‑C) and confirmation of ≥2 such episodes (Q4‑E), distinguishing recurrent from single/isolated attacks.

Breathlessness attributable to cardiopulmonary disease: Assessed via the exclusionary skip‑item at Q5‑A, which routes participants whose walking disability is due to a non‑cardiopulmonary condition out of the breathlessness symptom series, isolating heart/lung‑attributable breathlessness in Q5‑B–F.

Composite "Chronic Respiratory Symptoms" outcome: A derived (non‑native) variable classifying a participant as symptom‑positive if any one of the above chronic/recurrent constructs (chronic cough, chronic phlegm, recurrent exacerbation, recurrent wheeze/attack) was positive; symptom‑negative otherwise. This composite is an analytic construction and does not correspond to a single ATS‑DLD‑78‑A item.

Table 2. Operational Definitions Used in the Study

| **S. No.** | **Disease** | **Definitions** |
| --- | --- | --- |
| 1. | **Silicosis** | An occupational, fibronodular lung disease caused by inhalation of dust containing crystalline silica (quartz/silicon dioxide), pathologically marked by progressive fibrotic nodules in the lung parenchyma(Adamcakova & Mokra, 2021).  Chronic silicosis is recognized in two forms. Simple (or nodular) chronic silicosis is characterized by a development of small (<1 cm in size) and hard nodules in the upper lung lobes. These patients may be asymptomatic or may have dry cough or exertional dyspnoea(Adamcakova & Mokra, 2021). |
| 2. | **Pneumoconiosis** | Pneumoconiosis is any lung disease caused by the inhalation of organic or nonorganic airborne dust and fibers. Patients usually encounter these inhalants in the workplace environment, and therefore it is known as an occupational disease. The most frequently encountered types of pneumoconiosis are asbestosis, silicosis, and coal miner’s lung. These particles cause inflammation and fibrosis in the lung resulting in irreversible lung disease.(MR Farzaneh, 2010) |
| 3. | **Progressive Massive Fibrosis (PMF)** | Progressive massive fibrosis (PMF) is a severe form and late-stage chronic pneumoconiosis defined by the clustering of silicotic nodules fused with connective tissue in silicosis and coal macules surrounded by fibrous tissue in coal workers’ pneumoconiosis.(Deivasigamani et al., 2024) |
| 4. | **Crystalline silica / Respirable crystalline silica (RCS)** | Respirable crystalline silica (RCS) refers to the silica in crystalline form, such as quartz or cristobalite, that is present in respirable dust and can pose health risks in occupational environments, particularly in traditional ceramics industries.(Monfort et al., 2014) |
| 5. | **Chronic Respiratory Disease (CRD) symptoms** | CRDs are chronic diseases of the airways and other structures of the lung. Some of the most common are asthma, COPD, respiratory allergies, occupational lung diseases and pulmonary hypertension.(World Health Organization, 2026) |
| 6. | **Silico-tuberculosis** | TB in this article refers to thoracic disease due to M. tuberculosis and excludes latent TB infection (LTBI). Silicotuberculosis is defined as the combination of silicosis and active TB and/or post-tuberculous lung disease (PTLD).(Ehrlich et al., 2024) |

**Abbreviations Used in the Manuscript**

| **Abbreviation** | **Full Form** |
| --- | --- |
| CRDs | Chronic Respiratory Disease |
| ATS-DLD | American Thoracic Society – Division of Lung Disease (questionnaire) |
| OR | Odds Ratio |
| CI | Confidence Interval |
| SPSS | Statistical Package for the Social Sciences |
| PPE | Personal Protective Equipment |
| TB | Tuberculosis |
| ATT | Anti-Tuberculosis Treatment |
| ICMR | Indian Council of Medical Research |
| IEC | Institutional Ethics Committee |
| PMF | Progressive Massive Fibrosis |
| RCS | Respirable Crystalline Silica |
| SD | Standard Deviation |
| INR | Indian Rupee |
| n | sample size / sub-group frequency |
| km | Kilometre |
| h | Hour(s) |
| p | Probability value (statistical significance) |
